# Long noncoding RNA UCA1 induced by SP1 promotes cell proliferation via recruiting EZH2 and activating AKT pathway in gastric cancer

**DOI:** 10.1038/cddis.2017.143

**Published:** 2017-06-01

**Authors:** Zhen-Qiang Wang, Qiang Cai, Lei Hu, Chang-Yu He, Jian-Fang Li, Zhi-Wei Quan, Bing-Ya Liu, Chen Li, Zheng-Gang Zhu

**Affiliations:** 1 Shanghai Key Laboratory of Gastric Neoplasms, Department of Surgery, Shanghai Institute of Digestive Surgery, Ruijin Hospital, Shanghai Jiao Tong University School of Medicine, Shanghai, China; 2 Department of General Surgery, XinHua Hospital, Shanghai Jiao Tong University School of Medicine, Shanghai, China

## Abstract

Long noncoding RNA UCA1 has emerged as a novel regulator in cancer initiation and progression of various cancers. However, function and underlying mechanism of UCA1 in the progression of gastric cancer (GC) remain unclear. In the present study, we report that UCA1 expressed highly in GC tissues and GC cells, which was partly induced by SP1. UCA1 promoted GC cell proliferation and G1/S transition *in vitro* and *in vivo*. Moreover, UCA1 exerted its function through interacting with EZH2, promoting direct interaction with cyclin D1 promoter to activate the translation of cyclin D1. Furthermore, AKT/GSK-3B/cyclin D1 axis was activated to upregulate cyclin D1 due to overexpression of UCA1. In addition, EZH2 and phosphorylated AKT induced by UCA1 could impact each other to form a positive feedback to promote cyclin D1 expression. This study demonstrated that UCA1 as a critical regulator involved in GC proliferation and cell cycle progression by promoting cyclin D1 expression, which indicates that it may be clinically a potential therapeutic target in GC.

Gastric cancer (GC) has been a heavy health burden worldwide especially in eastern Asia because of high incidence rates and mortality rates, which is the third leading cause of tumor death.^1^ Although a steady decrease in GC incidence has been noted, early-stage diagnosis and late-stage comprehensive effective treatment still are the main shortnesses for GC therapy.^2^ Novel molecular mechanisms accounting for the initiation and progression of GC should be investigated to better figure out the way to prevent and cure the disease.

With the advent of the human genome sequence technology, noncoding RNAs have been researched and considered as a new breakthrough to gain a better understanding of the initiation and progression of cancers.^3, 4^ Recently, there has been highly interest in uncovering the underlying mechanisms of long noncoding RNAs (lncRNA) in different types of cancer.^5^ Long noncoding RNAs, RNA molecules longer than 200 nucleotides, have a vital role in gene regulation and cell function.^6^ Major attention was attracted to long noncoding RNAs, thanks to its crucial role involved in some molecular events, which usually promote the progression of cancer. More and more evidences demonstrated that dysregulation of lncRNAs could be a novel gap to understand the misbehavior of cancer in proliferation metastasis and metabolism.^7, 8^ LncRNAs could have multi-roles including oncogenes or tumor suppressor in regulating the initiation and progression of cancer.

Urothelial cancer associated 1 (UCA1) was initially discovered and investigated in bladder cancer, which has oncogenic roles in tumor proliferation and metastasis.^9, 10^ The expression of UCA1 has been reported to be upregulated in other cancers, including breast cancer, colorectal cancer, ovarian cancer, hepatocellular carcinoma and GC.^11, 12, 13, 14, 15^ Some transcription factors such as Ets-2, CCAAT/enhancer-binding protein *α* have been reported to bind the core promoter of UCA1 to enhance its expression.^16, 17^ UCA1 was upregulated in GC and associated with differentiation, TNM stage and poor prognosis, which was reported in recent study. However, the mechanism of UCA1 about proliferation and other biology behaviors of GC is not investigated.

In this study, we mainly focus on the influence of UCA1 on GC cells and the underlying mechanism of UCA1 in gastric cancer. The aim of the study was to figure out the following questions: (i) expression level of UCA1 in GC and the role in GC; (ii) the reason accounting for upregulation of UCA1 in GC; (3) potential downstream target and pathway of UCA1 involved in proliferation in gastric cancer.

## Results

### Overexpression of UCA1 and clinical characteristics

**Figure 1:**
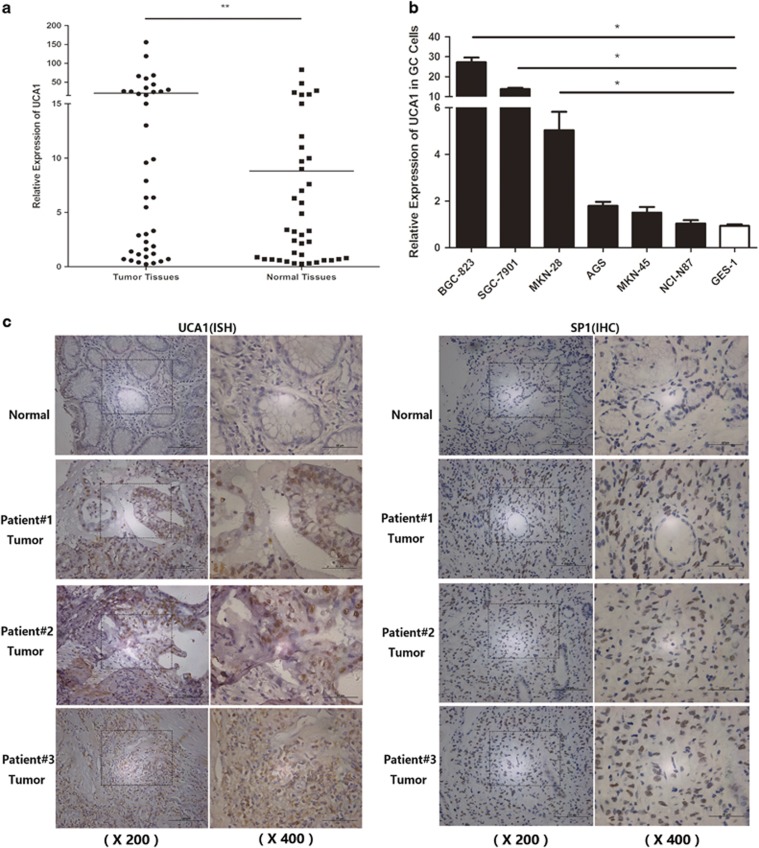
Overexpression of UCA1 in GC. (**a**) The expression of UCA1 in 39 GC tissues and matched normal samples were detected using quantitative reverse transcription PCR. The statistical differences between the samples were analyzed with paired samples *t*-test (*n*=39, *P*<0.01). (**b**) UCA1 expression in GC cell lines including BGC-823, SGC-7901, AGS, MKN-45, MKN-28, NCI-N87 and GES-1. The expression of UCA1 was normalized to that in GES-1. The statistical differences between groups were analyzed using independent samples *t*-test. Error bars represent the mean±S.D. of triplicate experiments (*P<0.05, **P<0.01). (**c**) Expression level and location of SP1 and UCA1 in GC tissues and normal samples. Distribution of UCA1 is both in the cell nucleus and the cytoplasm

First, the UCA1 expression in tumor tissues and its matched nontumor tissues of 39 patients with GC was measured by real-time RT-PCR. We found that UCA1 expression level was significantly higher in tumor tissues than that in nontumor tissues. Among the 39 GC tissues, 69.2% (27/39) of the tumor samples showed upregulation of UCA1 compared with the matched nontumor tissues (relative expression ratio <1.0, *P*<0.01, Figure 1a). Furthermore, 56.4% (22/39) of GC samples showed increased expression of UCA1 above the twofold cutoff (relative expression ratio <0.5). Then, we investigated the association of UCA1 expression with clinical characteristics: overexpression of UCA1 indicated higher tumor stage and lymph node metastasis in GC patients. The rest of analyzed factors showed no significant correlation (Table 1). Next, six different kinds of GC cell lines including BGC-823, SGC-7901, AGS, MKN-45, MKN-28, NCI-N87 and immortalized gastric epithelium cell GES-1 were also used to detect the expression level of UCA1. Major GC cell line presented markedly higher expression than GES-1 (Figure 1b). Subsequently, *in situ* hybridization (ISH) was used to detect and locate the UCA1 in GC tissues and normal samples. GC tissues presented much stronger UCA1-positive staining than normal tissues. In addition, UCA1 gathered both in the cytoplasm and nuclear region (Figure 1c left panel).

### UCA1 upregulated by transcription factor SP1

**Figure 2:**
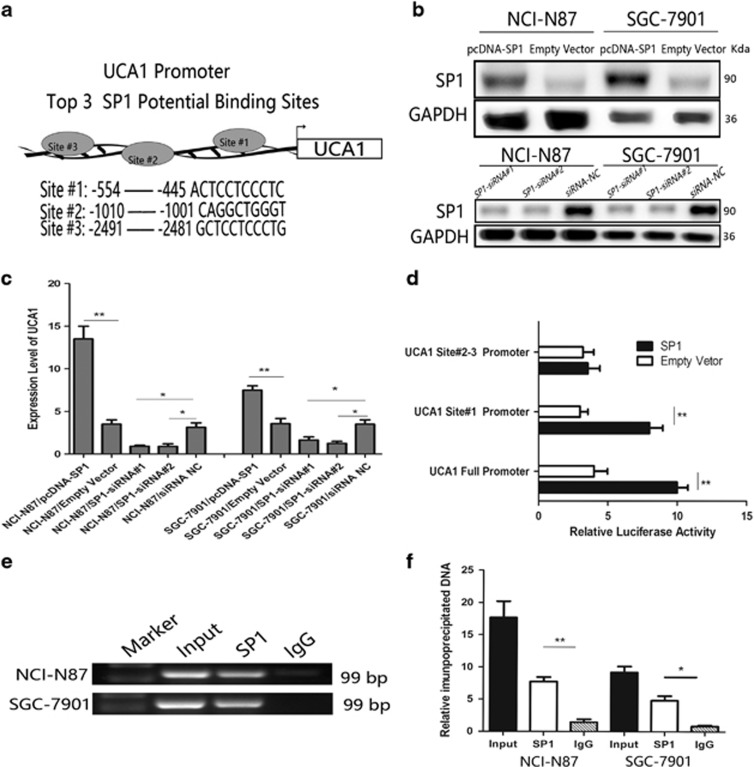
The transcription factor SP1 is involved in UCA1 upregulation. (**a**) The predicted top three positions of putative SP1-binding sites in −2.5 kb human UCA1 promoter from JASPAR database. (**b**) Upregulation of SP1 and slicing of SP1 by transfecting pcDNA-SP1 and SP1 siRNAs, respectively, which were determined by western blot in the protein expression level. (**c**) RT-PCR was used to determine the expression of UCA1 in cells transfected with pcDNA-SP1 and SP1 siRNAs, respectively. Error bars represent the mean±S.D. of triplicate experiments (*P<0.05, **P<0.01). (**d**) A dual-luciferase reporter assay was performed by co-transfecting the full-length UCA1 promoter (full promoter), UCA1 promoter only containing binding site #1 promoter (Site #1) or the rest of UCA1 promoter fragment (Site #2 and 3) with SP1 expression vector or an empty vector in NCI-N87 cells. Luciferase activity was expressed as relative to that of the pGL3-blank-vector. Error bars represent the mean±S.D. of triplicate experiments (**P<0.01). (**e** and **f**) CHIP assay was performed to determine the interaction between SP1 and binding site #1 of UCA1 promoter. Anti-SP1 significantly harbored UCA1 promoter than anti-IgG by using RT-PCR analysis. Error bars represent the mean±S.D. of triplicate experiments (*P<0.05, **P<0.01)

To investigate which transcript factor upregulated UCA1 in GC, the JASPAR CORE database was used to analyze the promoter of UCA1. The top three binding sites in UCA1 promoter were listed in Figure 2a. As the site #1 (−554 to approximately −445) was closed to the core promoter of UCA1, which indicated that the site #1 may be the right binding site. So we selected SP1 as the priority candidate transcript factor.

We found that UCA1 expression levels were significantly elevated in NCI-N87 and SGC-7901 cells when SP1 was overexpressed by transfected pcDNA-SP1, whereas UCA1 expression was downregulated when SP1 was knocked down by transfecting SP1 siRNAs in NCI-N87 and SGC-7901 cells (Figures 2b and c). To further confirm the results, we constructed three luciferase report vectors containing full length of UCA1 promoter, −800 to −100 bps (site #1) of UCA1 promoter and −800 to −2500 (site #2 and 3) of the UCA1 promoter, respectively. As shown in Figure 2d, SP1 could significantly increase the luciferase activity in NCI-N87 cells by transfecting the full UCA1 promoter vector and containing site #1 vector, respectively, compared with the control cells. In addition, the lack of site #1 binding sites impaired the luciferase activity, which suggested that SP1 could activate UCA1 transcription and site #1 binding site is crucial for regulation of SP1 on UCA1. Moreover, we performed chromatin immunoprecipitation (ChIP) assays with SP1 antibody to determine whether the site #1 region in the UCA1 promoter mediated SP1 binding to the endogenous UCA1 promoter. Both in NCI-N87 and SGC-7901 cells, SP1-prompter binding activity was detected and significantly higher than IgG control group (Figures 2e and f). Furthermore, immunohistochemistry was used to detect SP1 in same GC patients’ paired tumor and normal tissues. SP1 in GC tissues also presented stronger positive staining than normal tissues as UCA1 (Figure 1c, left panel). These results indicate the possibility that UCA1 overexpression in GC is mediated by the SP1 transcription factor.

### Promotion of UCA1 on GC cell proliferation and cell cycle

**Figure 3:**
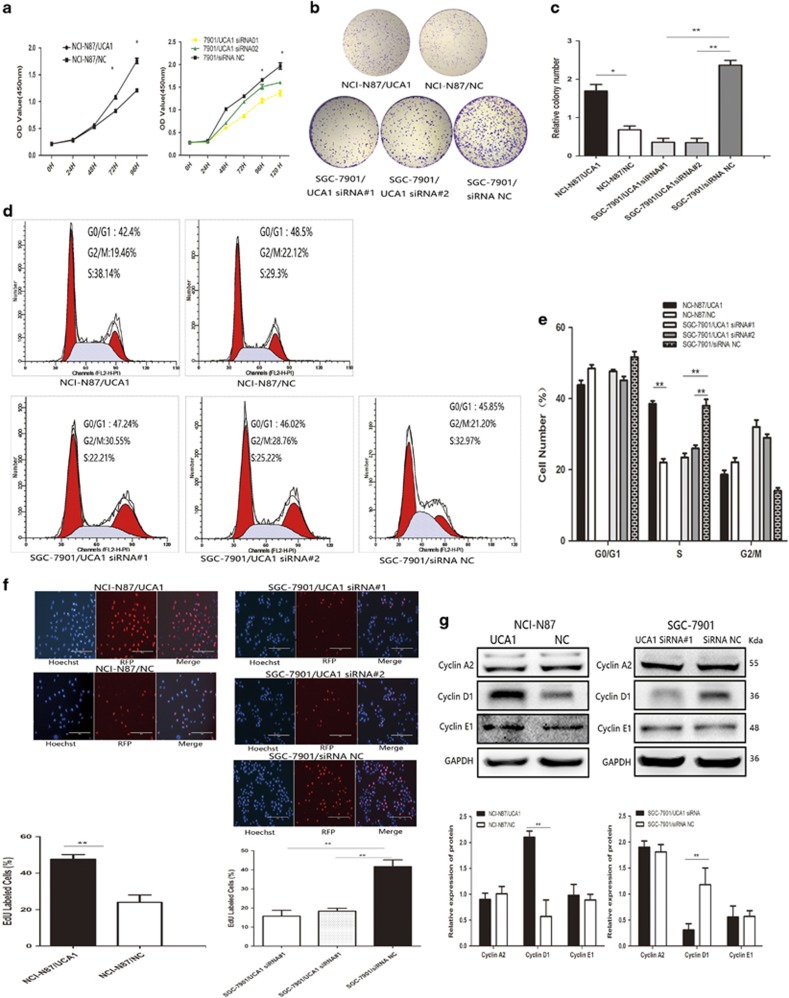
Effects of UCA1 on GC proliferation and cell cycle *in vitro*. (**a**) CCK-8 assay was performed to determine the effect of overexpression of UCA1 by transfecting pcDNA-UCA1 to NCI-N87 cell and knockdown UCA1 via transfecting UCA1 siRNAs in SGC-7901 cells. UCA1 considerably promoted GC cell growth (*P<0.05). (**b** and **c**) UCA1 increased colony-formation ability in NCI-N87 cells and slicing of UCA1 led to inhibition of colony-formation ability in SGC-7901. Error bars represent the mean±S.D. of triplicate experiments (*P<0.05, **P<0.01). (**d** and **e**) UCA1 increased the cells in S phase compared with negative control in NCI-N87 cells and UCA1 siRNAs remarkably decreased the ratio of cells in S phase and led to G0/G1 arrest in SGC-7901. Error bars represent the mean±S.D. of triplicate experiments (**P<0.01). (**f**) Overexpression UCA1 in NCI-N87 cells promoted more cells into S phase than negative control via performing EdU dye assay. Slicing UCA1 in SGC-7901 hardly led to an accumulation of cells in S phase than negative control (**P<0.01). (**g**) Effects of overexpression or downexpression of UCA1 on protein involved in cell cycle. UCA1 elevated the expression of cyclin D1 expression in NCI-N87 cell. Downregulating UCA1 inhibited the expression of cyclin D1. Cyclin A2 and cyclin E1 showed indifference. Error bars represent the mean±S.D. of triplicate experiments

To investigate the biological function of UCA1 in GC, we set up UCA1 overexpression cell, NCI-N87/UCA1, by transfecting pcDNA-UCA1 into NCI-N87, and set up UCA1 knockdown expression cell, SGC-7901/siUCA1, by transfecting UCA1 siRNA into SGC-7901. To better understand the function of UCA1 in GC, MKN-28 cell line was also selected to perform gain-and-loss experiments, which were presented in the Supplementary Information. Cell-proliferation assay (CCK-8) showed that cell proliferation of NCI-N87/UCA1 was significantly increased. Although SGC-7901/siUCA1#1 and siUCA1#2 were observably inhibited compared with its control cells (Figure 3a, *P<0.05), the NCI-N87/UCA1 cells had significantly increased growth and proliferation of GC cells. Meanwhile, SGC-7901/UCA1-siRNA1 and siRNA2 manifested observably growth inhibition compared with control cells (Figure 3a, *P<0.05). Consistent with CCK-8 assay results, the number of cell colony formation was markedly increased in the NCI-N87/UCA1 cells and fewer cell colony formation was detected in the SGC-7901/siUCA1 cells (Figures 3b and c, *P<0.05; **P<0.01). The proliferation of MKN-28 cells was also improved via upregulating UCA1 expression (Supplementary Figures 1a and b, *P<0.05). Given that upregulated UCA1 increased the GC cell growth, we sought to determine its cell cycle progression via flow cytometric analysis. As shown in Figures 3d and e (**P<0.01), UCA1 overexpression promoted NCI-N87 cell cycle progression and the population of cells in the S phase was significantly increased. Moreover, decreased S phase was detected in SGC-7901/siUCA1 compared with its control cells. In MKN-28 cell lines, similar results were also detected (Supplementary Figures 2a and b *P<0.05).

To further determine whether UCA1 could affect GC cell cycle progression, we performed EdU dye assay to detect the ratio change of cells entering the S phase. The results revealed that more NCI-N87/UCA1 cells entered S phase than its control cells; on the contrary, the SGC-7901/siUCA1 cells that entered S phase were significantly less than its control cells. (Figures 3f, **P<0.01). In MKN-28 cell lines, similar results were also detected (Supplementary Figures 2c and d *P<0.05). In addition, the proteins involved in cycle progression were examined by using western blot. The results showed that cyclin D1 was significantly increased in the NCI-N87/UCA1 cells and decreased cyclin D1 was detected in SGC-7901/siUCA1 cells compared with its control cells. (Figure 3g). Cyclin D1 is one of the most important regulators in G1/S transition. However, the expression of cyclin A2 and cyclin E1 showed no difference. Taken together, UCA1 could promote GC proliferation and impact cycle progression via affecting G1/S transition.

### UCA1 promotes the G1/S transition of GC cells through cyclin D1

**Figure 4:**
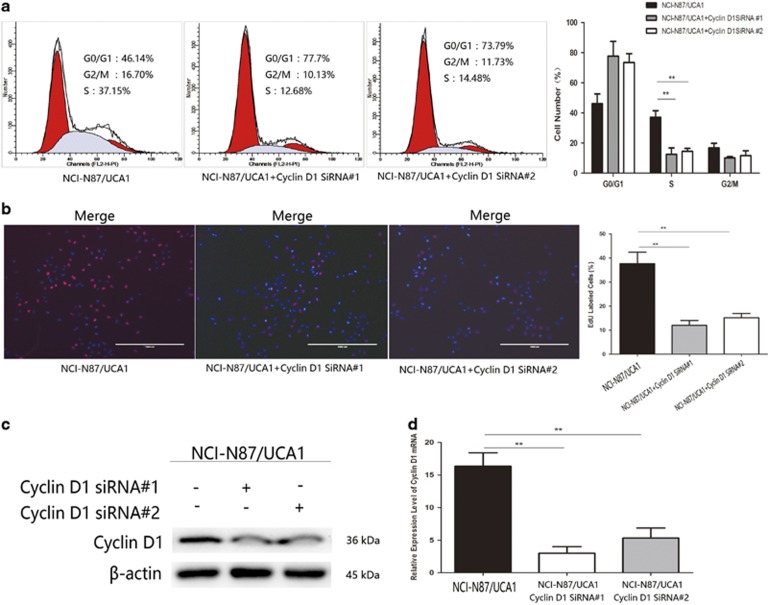
Cyclin D1 mediated the regulation of UCA1 on G1/S transition. (**a**) NCI-N87 cells co-transfected pcDNA-UCA1 and cyclin siRNAs showed significantly decreased ratio of cell in S phase than NCI-N87 cell with pcDNA-UCA1 alone and accumulation of cells in G0/G1 phase (**P<0.01). (**b**) EdU dye assay was performed to further determine the inhibition of G1/S transition in NCI-N87 cells co-transfecetd with pcDNA-UCA1 and cyclin D1 siRNAs. Cylin D1 siRNAs significantly reverse the regulation of UCA1 on G1/S transition in NCI-N87 (**P<0.01). (**c** and **d**) Western blot and qRT-PCR were used to determine the effect of cyclin D1 on NCI-N87 cell transfected with pcDNA-UCA1. Cylin D1 siRNAs inhibited the expression of cyclin D1 both in mRNA and protein level to partly reverse the modulation of UCA1 on cyclin D1 in NCI-N87 cell (**P<0.01). Error bars represent the mean±S.D. of triplicate experiments

To explore whether cyclin D1 mediated regulation of UCA1 on GC cell G1/S transition, we co-transfected cyclin D1 siRNAs and pcDNA-UCA1 plasmid into NCI-N87 cell and analyzed the distribution of cells in different phases by flow cytometry. The increase of S phase ratio by increasing UCA1 was reversed in part by co-transfected cyclin D1 siRNAs, which also led to G1 phase arrest (Figures 4a, **P<0.01). Consistently, EdU dye assay results demonstrated that co-transfection of cyclin D1 siRNAs could partly inhibit the function of UCA1 on increasing GC cells G1/S transition (Figures 4b, **P<0.01). The rescue performance was also carried out in MKN-28 cells, cyclin D1 siRNA could block the G1/S transition induced by increased UCA1 (Supplementary Figures 3a and b, *P<0.05). Furthermore, cyclin D1 mRNA and protein expression were detected by real-time RT-PCR and western blot, respectively. Co-transfected cyclin D1 siRNAs could inhibit upregulation of cyclin D1 resulted from overexpression of UCA1 in both mRNA and protein level (Figures 4c and d, **P<0.01). Similar results were found in MKN-28 cell line (Supplementary Figure 3c, *P<0.05). Taken together, these results suggest that UCA1 could control G1/S transition by affecting cyclin D1 expression.

### UCA1 promotes cyclin D1 expression via increasing EZH2 and activating AKT pathway

**Figure 5:**
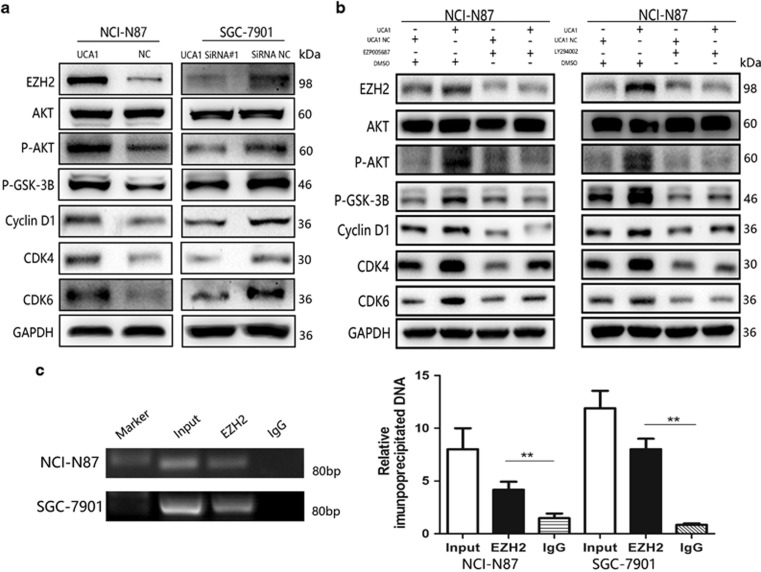
EZH2 and AKT pathway is involved in regulation of UCA1 on cyclin D1 expression. (**a**) EZH2 and AKT/GSK-3B/ cyclin D1 axis-related proteins were detected by immunoblotting, using extracts from the NCI-N87 transfected with pcDNA-UCA1 and SGC-7901 with UCA1 siRNAs and negative control cell, respectively. GAPDH is used as loading control. Overexpression of UCA1 in NCI-N87 cells significantly increased EZH2 expression and activated the AKT/GSK-3B/cyclin D1 axis. Downregulating UCA1 in SGC-7901 cells inbibited the activations of these proteins. (**b**) EZH2-specific inhibitor EZP005687 and P-AKT inhibitor LY294002 were used to culture NCI-N87 cells transfected with pcDNA-UCA1 and empty vectors. Effect of EZP005687 on NCI-N87 with pcDNA-UCA1 reflected in reduction of EZH2 and inactivation of AKT/GSK-3B/cyclin D1 and LY294002 showed the same effect on NCI-N87 cells with pcDNA-UCA1 compared with NCI-N87 cells with empty vectors. (**c**) CHIP assay was performed to determine the interaction between EHZ2 and cyclin D1 promoter. Anti-EZH2 significantly harbored more promoter fragments of cyclin D1 than anti-IgG both in NCI-N87 and SGC-7901 cells (**P<0.01). Error bars represent the mean±S.D. of triplicate experiments

Enhancer of Zeste Homologue 2 (EZH2) has a crucial role in gene expression regulation, which may vary depending on the cellular context. One-fifth of all human lncRNAs identified is physically associated with polycomb repressive complex 2 (PRC2, EZH2, SUZ12 and EED), and EZH2 is an important component of PRC2.^18, 19^ Recent studies have shown that EZH2 could promote cell cycle progression via affecting AKT pathway or cyclin expression and that EZH2 also be modulated by post-translational modifications through phosphorylation by AKT.^20, 21, 22, 23^ The complex regulation network triggered us come up with an assumption that EZH2 and AKT pathway may involve in regulation of UCA1 on cyclin D1 expression. First, we examined the EZH2, AKT, GSK-3B and cyclin D1 relevant cyclin-dependent kinase protein expression by western blot. As shown in Figure 5a, expression of EZH2 and phosphorylation-AKT (p-AKT) were markedly higher in the NCI-N87/UCA1 cells than that in negative control cells. And as the direct downstream of AKT protein, phosphorylation-GSK-3B (p-GSK-3B) was also upregulated so that the inhibition on cyclin D1 was significantly reduced. In addition, CDK4 and CDK6 expression were also upregulated. On the other hand, the SGC-7901/siUCA1 cells showed opposite results (Figure 5a). Next, we used EZH2 inhibitor (EZP005687)- and p-AKT inhibitor (LY49002)-treated NCI-N87/UCA1 cells, respectively and found that EZH2 inhibitor (EZP005687) could markedly inhibit EZH2 protein in NCI-N87/UCA1 cells and also decreased the expression of p-AKT and its downstream target p-GSK-3B, which led to the reduction of cyclin D1 significantly following with attenuation of CDK4 and CDK6 (Figure 5b, left panel). On the other side, p-AKT inhibitor (LY49002) could also inhibit AKT pathway protein, which impaired expression of cyclin D1, CDK4 and CDK6. Meanwhile, EZH2 protein expression was downregulated in the NCI-N87/UCA1 cells treated with p-AKT inhibitor (LY49002; Figure 5b, right panel). These results indicated that UCA1 promotes cyclin D1 expression via affecting EZH2 and activating AKT pathway and that EZH2 and AKT could induce mutual effects on each other in GC cell. Beyond that, EZH2 was reported that which could directly interact with the promoter of cyclin D1.^20^ Here we performed CHIP assay to determine whether EZH2 could bind to cyclin D1 to activate cyclin D1 translation in GC cell. As shown in Figure 5c, EZH2 antibody could significant harbors more cyclin D1 promoter DNA fragment than IgG antibody in both NCI-N87 cells and SGC-7901 cells (Figure 5c, **P<0.01). In MKN-28 cell lines, similar results were also detected (Supplementary Figures 3d and e, *P<0.05). Taken together, these findings indicated that UCA1 could increase cyclin D1 expression by promoting EZH2 and through AKT pathway. EZH2 could activate AKT pathway and be promoted by AKT pathway. Furthermore, EZH2 could increase cyclin D1 translation by binding to the promoter of cyclin D1.

### Physical interaction between UCA1 and EZH2 in gastric cancer cell

**Figure 6:**
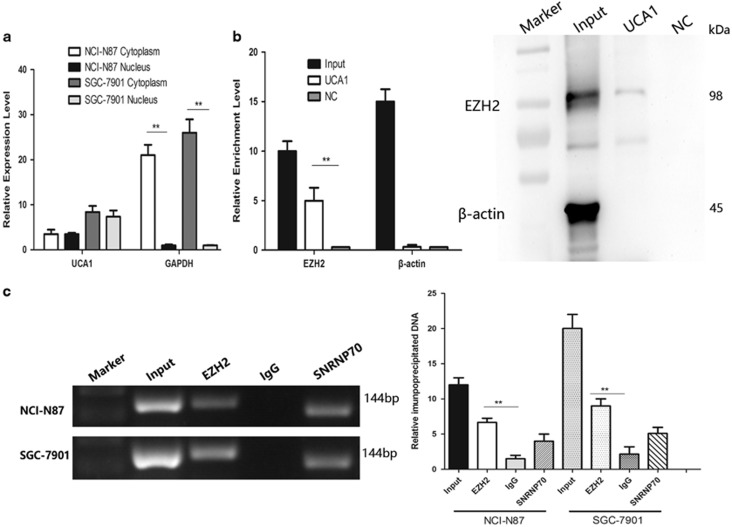
UCA1 interacts with EZH2 in GC. (**a**) Distribution of UCA1 in GC was determined by qRT-PCR after isolating nucleus RNA and cytoplasm RNA. UCA1 are both localized in nucleus and cytoplasm. (**b**) RNA pull-down assay was performed to determine the association between UCA1 and EZH2. UCA1 RNA-bound EZH2 protein was detected via western blot and no band showed up in negative control (**P<0.01). *β*-actin acted as the loading control. (**c**) RIP assay was used to further confirm the interaction between UCA1 and EZH2. Anti-EZH2 significantly harbored more UCA1 fragments than anti-IgG via RT-PCR (**P<0.01). Error bars represent the mean±S.D. of triplicate experiments. Anti-SNRNP70 was used as a positive control

EZH2 is a crucial component of PRC2, which was reported physically associated with one-fifth of lncRNAs to date. We postulated that UCA1 may interact with and bind to EZH2 to regulate downstream molecular events in view of the regulation of UCA1 on EZH2 protein expression. To determine UCA1 localization in GC cells, UCA1 was detected by qRT-PCR with nucleus RNA and cytoplasm RNA (Figure 6a), and UCA1 in tumor tissue by ISH (Figure 1c). Next, interaction of UCA1 with EZH2 was determined by RNA pull-down assay, which revealed that biotin-labeled UCA1 could harbor EZH2 protein. And *β*-actin protein was not detected after biotin-labeled UCA1 precipitation, which suggested UCA1 could specially interact with EZH2 protein (Figures 6b, **P<0.01). To further confirm the assumption, RNA immunoprecipitation assay (RIP) was performed. We found that UCA1 was significantly enriched with the EZH2 antibody compared with IgG (negative control) in NCI-N87 cells and SGC-7901 cells and SNRNP70 was also detected as positive control (Figures 6c, **P<0.01). These results demonstrated that UCA1 could physically interact with EZH2.

### Effect of UCA1 on tumorigenesis of GC cell *in vivo*

**Figure 7:**
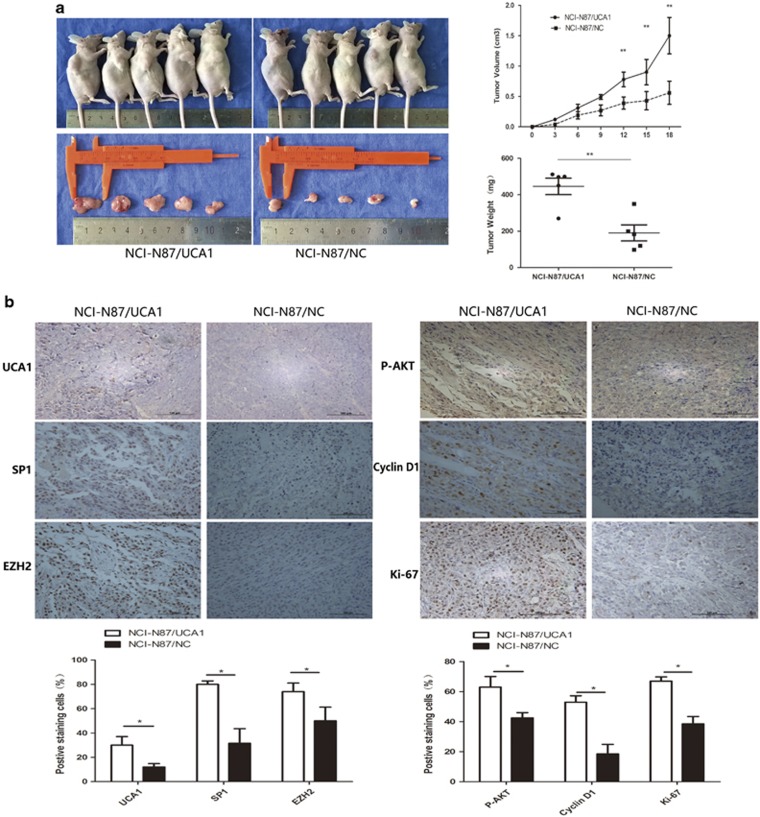
Effect of UCA1 on tumorigenesis *in vivo*. (**a**) Upregulating UCA1 in NCI-N87 via transfecting LV-UCA1 vectors increased the ability of tumorigenesis of NCI-N87, which reflected in the tumor weight and tumor volume (**P<0.01). (**b**) UCA1, SP1, EZH2, P-AKT, cyclin D1 and Ki-67 staining was performed to further determine the effect of UCA1 on cell proliferation. NCI-N87 cells with LV-UCA1 vectors showed significantly stronger positive than the negative control cells (*P<0.05)

Based on the results of UCA1 *in vitro* assay, we speculated that UCA1 might take an important part in tumorigenesis. First, we transfected lentiviral vector (LV)-GFP-UCA1 vector to NCI-N87 cell to acquire cells (NCI-N87/UCA1) that stably express UCA1 in high level. Next, NCI-N87/UCA1 and NCI-N87/NC were subcutaneously nude mice, respectively. Three weeks after injection, the tumors formed in NCI-N87/UCA1 group were significantly larger than those in NCI-N87/NC group (Figure 7a). The average tumor weight and tumor volume were markedly higher in the NCI-N87/UCA1 group than in the NCI-N87/NC group (Figure 7a, right panel, **P<0.01). Moreover, UCA1, SP1, EZH2, P-AKT, cyclin D1 and Ki-67-staining assay revealed that more positive cells were found in the NCI-N87/UCA1 group compared with the control group (Figures 7b, **P<0.01). These results suggest that overexpression of UCA1 promotes tumor formation *in vivo*.

## Discussion

LncRNAs have gained huge attention in recent years due to its aberrant expression in a large range of cancers and multiple roles in cancer initiation, progression and metastasis. LncRNAs could act as activators or inhibitors to participate in a variety of biological processes via interacting with DNAs, mRNAs, microRNAs or proteins.^24, 25, 26^ LncRNA UCA1 has been determined to be upregulated in multiple cancers including GC.^13, 27, 28^ Here in this study, we also found that UCA1 was significantly higher in GC tissues compared with matched nontumor tissues. And overexpression of UCA1 is associated with high lymph node metastasis rates and late TNM stage indicating UCA1 has an oncogenic role in GC and may act as a prognostic factor of advanced GC. Lots of studies revealed that regulation of lncRNAs in translational level is similar to common genes.^16, 29, 30^ In bladder cancer, Ets-2 and C/EBP*α* could promote UCA1 expression via binding to UCA1 core promoter.^16, 17^ To investigate the reason for high UCA1 expression in GC, bioinformatics databases including Jaspar database and starBase were used to screen for potential proto-oncogenic transcription factor. Finally, we focused on SP1 transcription factor because of our previous study involved in SP1 regulation in GC,^31^ and SP1 presented relatively higher score than other regulators according to analysis of bioinformatics databases. In addition, SP1 has been reported to regulate lncRNA TINCR in GC.^29^ SP1 could positively regulate UCA1 expression by using RT-PCR to detect the changes of UCA1 expression in transfected pcDNA-SP1 or siRNAs GC cells. Following dual-luciferase assay and CHIP assay, both determined that SP1 could physically interact with the promoter of UCA1. These essential results suggested that SP1 activates UCA1 translational expression to modulate UCA1 in GC. In addition, overexpression of UCA1 promoted GC cell proliferation and cell cycle progression. The ratio of cell in S phase was significantly increased after upregulating UCA1 in GC cell, as well as the expression of cyclin D1 protein. After siRNA knockdown of UCA1, we found opposite results that ratio of G0/G1 was elevated and reduction of cells in S phase was detected. Moreover, cyclin D1 protein, the crucial G1/S transition regulator, decreased significantly. These results suggest that UCA1 functions as an oncogene in GC and involves in regulation of G1/S transition. We sought to determine whether cyclin D1 mediated the regulation of UCA1 on G1/S transition in GC. To achieve this, NCI-N87 cell co-transfected pcDNA-UCA1, and cyclin D1 siRNAs were harvested to detect the cell distribution in different phases via flow cytometry. In the intervention of cyclin D1 siRNAs, UCA1 failed to increase cell cycle progression and most cells arrested in G0/G1 phase, which was same as the results of EdU dye assay. Western blot and real-time RT-PCR results also revealed that cyclin D1 siRNAs impaired upregulation of UCA1 on cyclin D1 in protein and mRNA level. Defection and imbalance of G1/S transition have been considered as an important factor to trigger the development of cancer, which partly could be attributable to abnormal upregulation of cyclin D1.^32, 33^ Taken together, UCA1 upregulates cyclin D1 to accelerate G1/S transition, which promoted the GC cell proliferation and cycle progression.

We sought to determine the underlying molecular mechanisms by which UCA1 regulated cyclin D1 in GC. Several studies indicated the latent regulatory mechanism about regulation of UCA1 on cyclin D1. EZH2 as a crucial component of PRC2 has been reported in some studies, which were involved in the regulation of cyclin D1.^20, 22, 23, 24, 25, 26, 27, 28, 29, 30, 31, 32, 33, 34^ Moreover, AKT/GSK-3B/cyclin D1 axis was the classic pathway involved in modulation of cyclin D1 in cancer cell.^35, 36^ Furthermore, EZH2 and AKT could influence expression of each other indicating that they constituted a positive loop.^21, 37, 38, 39^ In addition, one-fifth of all human lncRNAs identified is physically associated with EZH2.^18, 19^ Here, gain and loss of UCA1 assay in GC cell has been performed. Western blot results demonstrated that increased UCA1 could upregulate EZH2 and active AKT/GSK-3B/cyclin D1 pathway. On the contrary, slicing of UCA1 led to reduction of EZH2 and inactivation of AKT pathway. Not only that, regulation of UCA1 on increasing cyclin D1 could be rescued by EZH2-specific inhibitors (EZP005687) and P-AKT inhibitors (LY294002), respectively. And EZP005687 could also inhibit phosphorylation of AKT, whereas reduction of EZH2 was detected because of LY294002.

**Figure 8:**
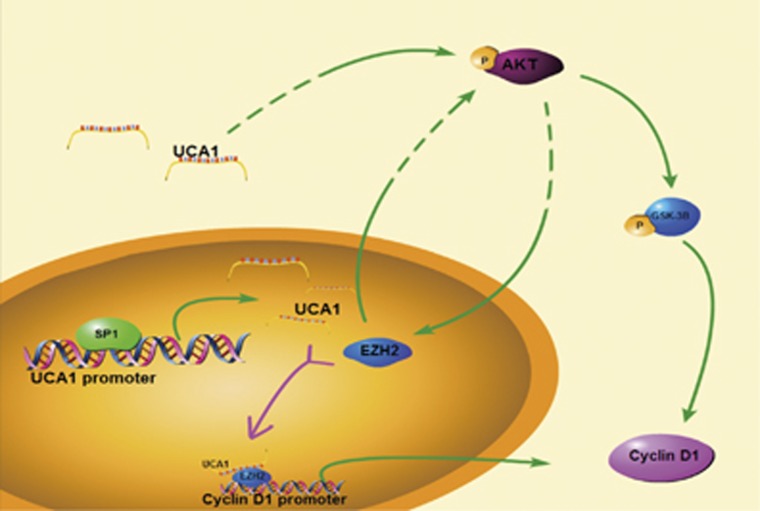
Schematic diagram illustrating signaling of EZH2 and its upstream activator and its downstream effectors in GC. SP1 directly binds to the core promoter of UCA1 so that it activates the expression of UCA1. UCA1 directly interacts with EZH2 and increase the expression of EZH2, which affects the activation of AKT/GSK-3B/cyclin D1 axis. EZH2 could also physically interact with the cyclin D1 promoter to promote the expression of cyclin D1. In addition, P-AKT could indirectly influence the expression of EZH2 so as to form a positive feedback loop with EZH2. With the accumulation of cyclin D1, G1/S transition was activated to promote cell proliferation and cancer progression

To determine the association between UCA1 and EZH2, RNA pull-down and RIP assay were performed. First, we detected EZH2-specific band, which harbored by biotin-labeled UCA1 RNA. There are three isoforms of UCA1 including 1.4, 2.2 and 2.7 kb. Here we focused on functional significance of 1.4 kb isoform of UCA1 because of its most abundant distribution in various cancers. Next, the results of RIP assay showed us that EHZ2 protein could bind to UCA1 RNA, which reflected the direct interaction between EZH2 and UCA1. In this view, direct physical interaction between EZH2 and UCA1 could partly explain the upregulation of EZH2 induced by overexpression of UCA1, which was also confirmed in the study by Hu *et al.*^40^ In addition, CHIP assay was performed to demonstrate that EZH2 could bind to the promoter of cyclin D1 to activate the translation process, which was also confirmed in the research by Yan *et al.*^20^ Comprehensive consideration of these results, UCA1 regulates cyclin D1 expression via interact directly with EZH2, which binds to promoter of cyclin D1 to active expression of cyclin D1 and brought mutual positive effect of EZH2 and AKT/GSK-3B/cyclin D1 axis (Figure 8). With the accumulation of dysregulated expression of cyclin D1, activation of CDK4 and CDK6 was also detected. At last, the biological property of UCA1 on promoting proliferation was also detected in the nude mice tumorigenesis assay. Ki-67 staining results also reflected that overexpression of UCA1 significantly increased growth and proliferation of GC cell.

In conclusion, the underlying molecular cross talk among UCA1, EZH2, AKT and cyclin D1, which is described here is believed to be the first reported in GC. UCA1 expression is significantly higher in GC tissues and might be a novel indicator for clinical application. UCA1 has a potent oncogenic role in regulating cell proliferation and cell cycle progression. However, limitations of this study still exist including lack of a large cohort of samples from patients with GC and short of survival analysis. It was reported that UCA1 could exert multiple functions in the development of cancer.^41^ Future studies of UCA1 on GC might be extended to the area, which could contribute to the treatment of GC.

## Materials and Methods

### Patients and GC cell lines

GC tissues and matched normal tissues were collected from 39 patients with GC in Ruijin Hospital between 2014 to 2015. The study was approved by the Human Research Ethics Committee of Ruijin Hospital, Shanghai Jiao Tong University, School of Medicine. Human GC cell lines BGC-823, SGC-7901, AGS, MKN-45, NCI-N87 and GES-1 were purchased from Shanghai Institutes for Biological Sciences, Chinese Academy of Sciences. MKN-28 cell line was purchased from American type culture collection (ATCC).

### RNA extraction and real-time PCR

Total RNAs were extracted with TRIzol reagent (Invitrogen, Carlsbad, CA, USA), nucleus and cytoplasm RNAs were isolated with RNeasy Midi Kit (Qiagen, Hilden, Germany). Real-time PCR analyses were conducted according to the manufacturer’s instructions (Life Technologies, Austin, TX, USA). Experiments were independently repeated in triplicate. The primers sequences have been listed in the Supplementary Information.

### RNA interference and vectors

Small interfering RNAs (siRNAs) specifically targeting human SP1, lncRNA UCA1 and cyclin D1 were purchased from GenePharma (Shanghai, China). The siRNAs were transfected into cells using the RNAiMAX reagent (Life Technologies) according to the manufacturer’s instructions. Vector pcDNA-SP1 as a gift came from Dr Hu (Ruijin Hospital, Shanghai, China). Vectors pcDNA-UCA1 and LV-GFP-UCA1 were brought from Genechem (Shanghai, China). Vector pcDNA-UCA1 and pcDNA-SP1 were transfected into cells via using the lipofectamine 2000 (Life Technologies). The RNA interference sequences were listed in the Supplementary Information.

### Cell-proliferation and cell cycle analysis

Cell viability was measured using Cell Counting Kit-8 (Dojindo, Japan). Cell cycle were analyzed by flow cytometry on a FACScan (Beckman Instruments, Fullerton, CA, USA). The cell-proliferation assays, colony-formation assays and cycle analysis were performed as described previously.^42^ Experiments were independently repeated in triplicate.

### Luciferase assay

Luciferase assays were performed using a luciferase assay kit (Promega, Madison, WI, USA) according to the manufacturer’s protocol, as previously described.^42^

### ChIP assay

ChIP assays were performed using the EZ ChIP Kit (Millipore, Billerica, MA, USA), according to the manual. Briefly, GC cells were cross-linked in 1% formaldehyde solution for 10 min at room temperature, followed by the addition of 125 mM of glycine for 5 min. DNA fragments ranging from 200 to 500 bp were yielded via sonication. Antibodies including anti-SP1, anti-EZH2 (Cell Signaling Technology, Danvers, MA, USA) and normal IgG were used for each immunoprecipitation. Immunoprecipitated and input DNAs were subjected to qRT-PCR analysis. The primers used for amplifying were listed in Supplementary Materials and Methods.

### RNA immunoprecipitation

We performed RNA immunoprecipitation (RIP) experiments using the Magna RIP RNA-binding protein immunoprecipitation kit (Millipore) according to the manufacturer’s instructions. Briefly, the cells were lysed in lysis buffer and cleared lysates were immunoprecipitated with indicated anti-EZH2 and IgG antibodies. Immunoprecipitated and input RNA were isolated and reverse transcribed. cDNA was used as a template in qRT-PCR amplifications with UCA1-specific primers. The primers used for amplifying were listed in the Supplementary Information.

### RNA pull-down

RNA pull-down assay were performed using Magnetic RNA-Protein Pull-Down Kit (Pierce, Rockford, IL, USA) according to the manual. Briefly, RiboMAX Large Scale RNA Production Systems (Promega) were used to yield full length of UCA1. The UCA1 RNA was bound to the beads to orient the RNA for protein binding after biotin labeling. RNA-bound beads were added into cell protein lysate for immunoprecipitation. The beads were washed and the samples were eluted with SDS-PAGE Loading Buffer for western blot analysis.

### Western blot

Western blot assays were performed as previously^42^ and the antigen–antibody reaction was visualized by enhanced chemiluminescence assay (ECL, Thermo, Rockford, IL, USA). The antibodies involved in the study were from Cell Signaling Technology. The experiments were independently repeated in triplicate.

### EdU dying

EdU dye assay was performed using Cell-Light EdU Apollo 567 *In Vitro* Imaging Kit (Ribobio Technology, Guangzhou, China) according to the manufacturer’s instructions. The labeled cells were counted under a microscope. The experiments were independently repeated in triplicate.

### Xenograft assay

Four-week-old male BALB/C nude mice were purchased from the Institute of Zoology, Chinese Academy of Sciences of Shanghai. All the experiments were performed in accordance with the official recommendations of the Chinese animal community. Two million NCI-N87 cells transfected with LV-GFP-UCA1 and LV-GFP-NC were injected subcutaneously in the upper flanks of mice, respectively. After 18 days, the mice were killed and the tumors were dissected.

### Immunohistochemistry

Immunohistochemical analysis was conducted as previously.^42^

### *In situ* hybridization

ISH was used to detect UCA1 in clinical specimens essentially based on a previously described method.^43^

### Statistical methods

Student’s *t*-test or one-way ANOVA were used for statistical analysis when appropriate. All statistical analyses were performed using SPSS 19.0 (SPSS Inc., Chicago, IL, USA). A two-tailed value of *P*<0.05 was considered statistically significant.

## Figures and Tables

**Table 1:** Association between UCA1 expression and clinical parameters of GC patients

	**UCA1 expression**		
**Clinical parameter**	**High group (*N*=22)**	**Low group (*N*=17)**	***P*-value (*χ***^**2**^**-test)**
*Age (years)*			
>60	17	10	0.226
<60	5	7	
			
*Gender*			
Male	14	10	0.767
Female	8	7	
			
*Tumor sizes*			
>5 cm	12	8	0.767
<5 cm	10	9	
			
*Tumor location*			
Distal	18	13	0.691
Proximal	4	4	
			
*Differentiation*			
Well to moderate	18	14	0.653
Poor	4	3	
			
*T stage*			0.226
T1+T2	2	4	
T3+T4	20	13	
			
*Lymph node metastasis*			
Negative	4	10	**0.008
Positive	18	7	
			
*TNM stages*			
I, II	3	8	*0.021
III, IV	19	9	

Based on relative expression ratios <0.5 (twofold cutoff), the 39 clinical cases were divided into two groups: low expression (*n*=17) and high expression (*n*=22). *P<0.05, **P<0.01
