# Supplementary figures and images for: Long noncoding RNA UCA1 induced by SP1 promotes cell proliferation via recruiting EZH2 and activating AKT pathway in gastric cancer
Source: Cell Death Dis. 2017 Jun 1;8(6):e2839–. doi: 10.1038/cddis.2017.143 (PMC5520878; doi:10.1038/cddis.2017.143)

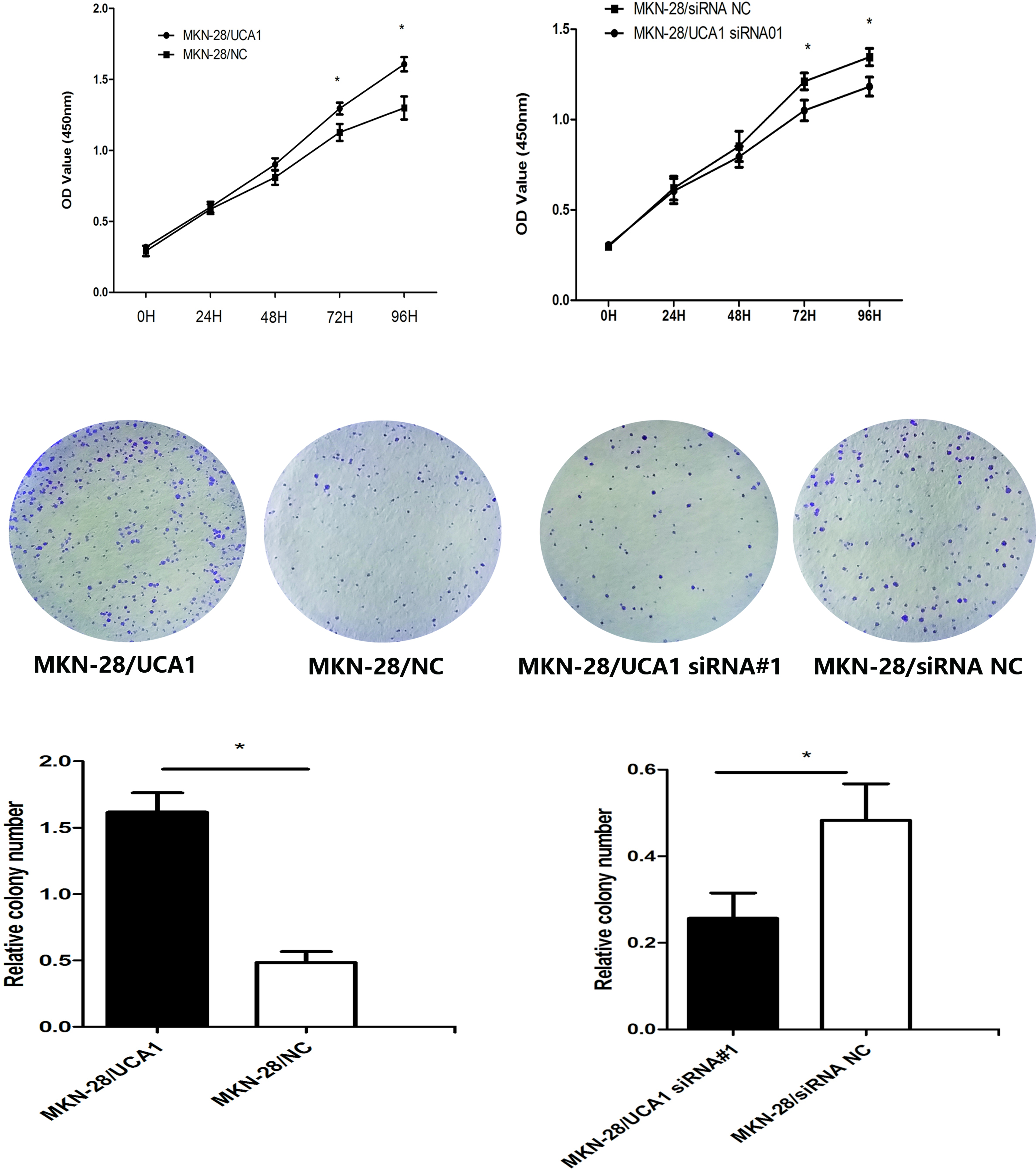

Supplement: Supplementary Figure 1 [file cddis2017143x1.tif]
