# Supplementary material for: Long noncoding RNA UCA1 induced by SP1 promotes cell proliferation via recruiting EZH2 and activating AKT pathway in gastric cancer
Source: Cell Death Dis. 2017 Jun 1;8(6):e2839–. doi: 10.1038/cddis.2017.143 (PMC5520878; doi:10.1038/cddis.2017.143)
Supplement: Supplementary Materials and Methods [file cddis2017143x4.doc]

supplementary materials and methods：

Primers sequences：

| UCA1 | Forward | 5'-CTCTCCATTGGGTTCACCATTC-3' | | |
| --- | --- | --- | --- | --- |
|  | Reverse | 5'-GCGGCAGGTCTTAAGAGATGAG-3' | | |
|  |  |  |  |  |
| Cyclin D1 | Forward | 5'-GCTGCGAAGTGGAAACCATC-3' | | |
|  | Reverse | 5'-CCTCCTTCTGCACACATTTGAA-3' | | |
|  |  |  |  |  |
| SP1 | Forward | 5'-TGGCAGCAGTACCAATGGC-3' | | |
|  | Reverse | 5'-CCAGGTAGTCCTGTCAGAACTT-3' | | |
|  |  |  |  |  |
| GAPDH | Forward | 5'-GGACCTGACCTGCCGTCTAG-3' | | |
|  | Reverse | 5'-GTAGCCCAGGATGCCCTTGA-3' | | |
|  |  |  |  |  |
| UCA1 promoter | Forward | 5'-AGGCTGGAGGCAGTGACGTC-3' | | |
|  | Reverse | 5'-TCCCAGCTACTCAGGAGGCTGAG-3' | | |
|  |  |  |  |  |
| Cyclin D1 promoter | Forward | 5'-GAACACCTATCGATTTTGCT-3' | | |
|  | Reverse | 5'-ACTGAATTCGTGAGCGTGAG-3' | | |
|  |  |  |  |  |
| UCA1 (RIP assay) | Forward | 5'-TGTTAGAGGGCTTGGGACAT-3' | | |
|  | Reverse | 5'-ATAGGTGTGAGTGGCGGTCT-3' | | |

RNA interference sequences：

| SP1 siRNA #1 | Forward | 5'-CCAACAGAUUAUCACAAAU-3' | | |
| --- | --- | --- | --- | --- |
|  | Reverse | 5'-GGUUGUCUAAUAGUGUUUA-3' | | |
|  |  |  |  |  |
| SP1 siRNA #2 | Forward | 5'-GGCUGGUGGUGAUGGAAUA-3' | | |
|  | Reverse | 5'-CCGACCACCACUACCUAU-3' | | |
|  |  |  |  |  |
| UCA1 siRNA #1 | Forward | 5'-GAGCCGAUCAGACAAACAATT-3' | | |
|  | Reverse | 5'-UUGUUUGUCUGAUCGGCUCTT-3' | | |
|  |  |  |  |  |
| UCA1 siRNA #2 | Forward | 5'-GGGCUUGGGACAUUUCACUTT-3' | | |
|  | Reverse | 5'-AGUGAAAUGUCCCAAGCCCTT-3' | | |
|  |  |  |  |  |
| Cyclin D1 siRNA #1 | Forward | 5-GCAUGUUCGUGGCCUCUAATT-3’ | | |
|  | Reverse | 5-UUAGAGGCCACGAACAUGCTT-3’ | | |
|  |  |  |  |  |
| Cyclin D1 siRNA #2 | Forward | 5'-CAAACAGAUCAUCCGCAAA-3' | | |
|  | Reverse | 5'-UUUGCGGAUGAUCUGUUG-3' | | |
|  |  |  |  |  |
| Negative control siRNA | Forward | 5-UUCUCCGAACGUGUCACGUTT-3 | | |
|  | Reverse | 5-ACGUGACACGUUCGGAGAATT-3 | | |
